# Examining the impact of land-use related factors on rural traffic collisions 

**Published:** 2019-08

**Authors:** Mahyar Vahedi Saheli, Meysam Effati

**Affiliations:** ^*a*^ Department of Civil Engineering, Faculty of Engineering, University of Guilan, Rasht, Iran.

## Abstract

**Background:**

More than 70% of fatal collisions belong to rural roads in Iran. A large number of these rural roads are no access control that allows the direct connection of land-uses to the main road. Direct connection of land-uses to the main road introduces conflicts and friction into the traffic stream that increases collision potential. This paper explores the Impact of area-based land-use variables on the frequency of traffic collisions on a divided rural multilane road.

**Methods:**

A case study of Rasht-Anzali road is chosen for this study. The collision data is obtained from the highway police department of Guilan province containing four years of collisions occurred in this road from 2014 to 2017. The land-use data is obtained from the rural guide plan of the villages locating beside the roadway supplemented by Google Earth maps. To develop the model inputs, the road centerline is buffered in both sides, and then the buffered roadway is divided into equal segments in each way due to the median dividing of the highway. The collision data has been divided into three different groups of collisions: vehicle-to-vehicle, pedestrian involved and all collisions. Count regressions are used to predict the frequency of collisions in road segments using land-use variables. With attention to over dispersion and excess zeros on the dependent variable, negative binomial and zero-inflated negative binomial regressions are used in this study. The best model for each group is chosen based on Vuong goodness-of-fit measure.

**Results:**

The results confirm some land-use impacts in previous researches and suggest some further implications for the interaction between land-uses and rural traffic collisions. With referring to models estimations, commercial, residential, government, and institutional land-uses played an important role in both vehicle-to-vehicle and pedestrian collisions, and religious land-use is found to have an increasing impact on pedestrian collisions.

**Conclusions:**

The findings can be useful in understanding the complex interrelationships between land-use and rural collisions, and in facilitating planners and policymakers to build safer roads.

**Keywords:**

Traffic collisions, Land-use, Road safety, Rural crash frequency, Count Regressions, Zero-inflated regressions

